# A case–control study of childhood leukaemia and paternal occupational contact level in rural Sweden

**DOI:** 10.1038/sj.bjc.6600113

**Published:** 2002-03-04

**Authors:** L Kinlen, J Jiang, K Hemminki

**Affiliations:** CRC Cancer Epidemiology Research Group, University of Oxford, The Radcliffe Infirmary, Oxford OX2 6HE, UK; Department of Biosciences at Novum, Karolinska Institute, 141 57 Huddinge, Sweden

**Keywords:** childhood leukaemia, Sweden, infection, paternal occupational contacts

## Abstract

In a national case–control study in Sweden, we investigated whether in rural areas (where susceptible individuals are more prevalent than in urban areas) leukaemia risk was higher among the young children of fathers with many work contacts, as the infective hypothesis has predicted. A total of 1935 cases diagnosed in 1958–1998 together with 7736 age-matched (within 1 year) population controls (of whom 970 and 3880 respectively were aged 0–4) were linked to paternal occupational details as recorded in the census closest to the year of birth. Applying the two classifications of occupational contact level used in a study of rural Scotland, the odds ratios for children aged 0–4 years in the highest contact category (which includes teachers) in the most rural Swedish counties were 3.47 (95% CI 1.54, 7.85) and 1.59 (1.07, 2.38) respectively, relative to the medium and low (reference) category; no such excess was found in urban or intermediate counties. There was also a significant positive trend at ages 0–4 in the rural counties across the three levels of increasing occupational contact (*P* for trend 0.02 and 0.03, respectively), but again not in the urban or intermediate counties. No such effect or trend was found at ages 5–14 in any of the three county groupings. The findings confirm those of a recent study in rural Scotland, and also suggest that unusual population mixing (as occurred in Scotland as a result of the North Sea oil industry) is not a necessary requirement for the effect, since comparable mixing has not been a feature of rural Sweden.

*British Journal of Cancer* (2002) **86**, 732–737. DOI: 10.1038/sj/bjc/6600113 www.bjcancer.com

© 2002 Cancer Research UK

## 

Significant excesses of childhood leukaemia have repeatedly been found in rural areas affected by marked population mixing (reviewed in Kinlen, 1995; Doll, 1999), most recently in a cohort study (Kinlen and Balkwill, 2001). Such rural ‘mixing’ situations would tend to promote epidemics of an underlying infection by increasing the level of contacts between susceptible and infected individuals, the former being more prevalent in rural areas. These findings therefore support the infective hypothesis of childhood leukaemia. Though the agent(s) remain to be identified, the absence of marked space–time clustering of the disease in the general population indicates that childhood leukaemia must be an uncommon response to the underlying infection – as it is in established infection-linked malignancies.

An additional approach to these largely geographic methods was first suggested by Kinlen and Stiller (1993), namely the study of risk in relation to parental occupational contact level. Compared to older school-attending children, pre-school children must in general have a more restricted range of frequent contacts with people outside the immediate family. An important contribution to the pool of infection to which pre-school children are exposed through their direct and indirect contacts must come from their fathers' work contacts, the level depending on the nature of that work. ‘High contact’ paternal occupations have already been associated with an increased incidence of childhood cytomegalovirus infection and paralytic poliomyelitis. An examination of this question within the excesses of childhood leukaemia associated with extreme rural population mixing found a greater risk among the children of men in high contact jobs (Kinlen, 1997). Also, a recent case–control study found a significant positive trend in risk among pre-school children across three levels of increasing paternal occupational contacts in rural Scotland (Kinlen and Bramald, 2001) with each of the two classifications of contact level developed in the 1997 study.

Because the findings in the Scottish study were more marked (though not significantly so) in a period of greater population mixing associated with the North Sea oil industry, a question was raised as to whether the latter was a necessary requirement, or whether the effects were typical of very rural areas *per se*. Sweden presents an example of a country, like Scotland, with a substantial rural population but without this type of population mixing. We have therefore examined childhood leukaemia risk in relation to paternal occupational contact level in a national Swedish case–control study, with particular reference to young children (0–4 years) in rural areas, applying the two classifications of contact level used in our two previous studies of the subject.

## MATERIALS AND METHODS

### Cases and controls

Cases and controls were selected from the Swedish Family-Cancer Database which links an administrative family register and census records to the Swedish Cancer Registry. The database holds selected details of all Swedes born after 1931, and of their parents, a total of 10.2 million individuals; it has been used in many previous studies (e.g. Hemminki and Mutanen, 2001; Hemminki, 2001). Updated in early 2001, it now includes details of cancers from the Swedish Cancer Registry for the years 1958–1998, and incidence rates can be derived that closely reproduce those recorded by that registry (Hemminki *et al*, 2001). Cases in the present study comprise all children (0–14 years) diagnosed with leukaemia in the years 1958–1998 for whom county of residence and a paternal occupation could be traced in the decennial census (1960, 1970, 1980 or 1990) closest to the year of birth. For each case, four controls with corresponding residential and paternal occupational details from the relevant census were randomly chosen from the database, matched on age (within 1 year), sex and county of residence.

### Paternal occupational contact levels

Paternal occupations in the database are coded to the two-digit Nordic Classification of Occupations, comprising 54 occupational groups (Andersen *et al*, 1999). These were allocated as far as was possible to the contact categories used in our previous studies (Kinlen, 1997; Kinlen and Bramald, 2001) in which a much larger number of different occupational titles were available for allocation to the categories by six independent advisors. Two separate (but partly overlapping) classifications were applied, as in the earlier studies. In the first of these, the categories were defined as follows:

- Very high contact: Children experience more new infections than any other age group and so would be expected to have an unusually high exposure to the infection postulated as underlying leukaemia. Since teachers, in turn, have prolonged and relatively close contact with children, they were regarded as representing a ‘very high’ contact category for present purposes.
- High contact: Other occupations that were judged to involve a greater than average level of contacts with many different people were service and professional workers such as sales workers, students, journalists, nurses, doctors, dentists and others who provide services directly to many different people; occupations involving appreciable contacts outside the local community (transport- and communication-related jobs), or regular changes of colleagues and work-place, as in the construction industry were also included here.
- Low, medium and indeterminate contact: All occupations not specified above (except ‘economically inactive’) formed this (reference) category. Children linked to fathers who were recorded as economically inactive in the relevant census were excluded from the study.

In the second classification of contact categories, certain occupations were moved from the ‘high’ to the ‘very high’ category (to join teachers), the reference category remaining unchanged. These included occupations that involve either frequent contacts outside the local community, namely transport- and communication-related jobs, or regular changes of colleagues and work-place, as in the construction industry. Except for the removal of these two groups of occupations, the high contact category remained as before.

The categories, by each classification, were defined in terms of the Nordic occupational codes before the leukaemia data were examined and they are given in more detail in the appendix.

### Rural–urban status

As the available dataset precluded a sensitive examination of communities by urban–rural status, the counties of Sweden were assembled into three groups of decreasing ‘rurality’, comprising: (i) the six most rural counties, including the five northernmost (population 1.09 million, density 4 per sq km); (ii) 15 intermediate counties (population 4.28 million, density 32 per sq km) and (iii) the four most urban counties, namely the cities of Stockholm (two counties), Gothenburg and Malmo (population 3.13 million, density 189 per sq km). Despite the marked differences in population density between the most rural and the intermediate groups of counties, the latter included substantial rural areas, while the most rural group held several towns, each with over 50 000 residents. The county groupings were defined before the leukaemia data were examined.

### Analysis

Using conditional logistic regression with EGRET software, matched data on cases and controls were examined within each of the three groupings of counties in relation to categories of paternal occupational contact level, as defined above. Tests for trends in risk across the contact categories were also carried out.

## RESULTS

Details of paternal county of residence and occupational contact category were available for a total of 1935 children diagnosed with leukaemia below age 15 in the period 1958–1998, together with 7736 age-matched controls. These cases represent a high proportion (about 84%) of all those diagnosed in Sweden in this period. Reasons for not including the remaining 16% of cases are absence of the father's name on the child's birth certificate, failure to identify the father's census record, paternal occupation not shown, father shown as economically inactive or county of residence missing in the census records.

For ages 0–4 years, the age group of primary interest, there were 970 cases and 3880 controls and for this group, the odds ratio for the highest contact category in the most rural counties was 3.47 (95% CI 1.54, 7.85) relative to the reference category. In these rural counties, there was also a significant positive trend across the three levels of increasing occupational contact (*P* for trend 0.02), but not in the urban and ‘intermediate’ counties, as shown in Table 1

**Table 1:**
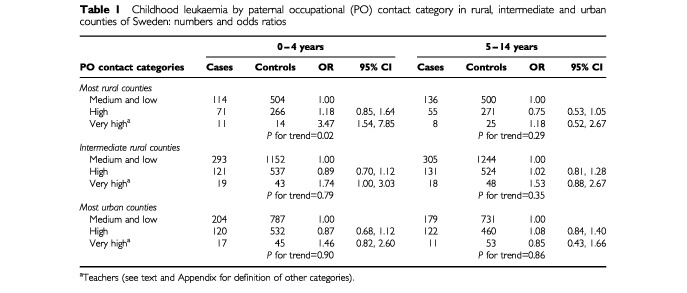
Childhood leukaemia by paternal occupational (PO) contact category in rural, intermediate and urban counties of Sweden: numbers and odds ratios

. In contrast, among the 965 cases and 3856 age-matched controls aged 5–14, the corresponding analyses showed no significant trend across the contact categories in either rural or the urban counties (Table 1).

Using the broader definition of the highest contact category, the excess risk at ages 0–4 years in the rural counties was less marked, though it remained significant (odds ratio 1.59; C.I 1.07, 2.38) and the positive trend across the categories persisted (*P*=0.03) as shown in Table 2

**Table 2:**
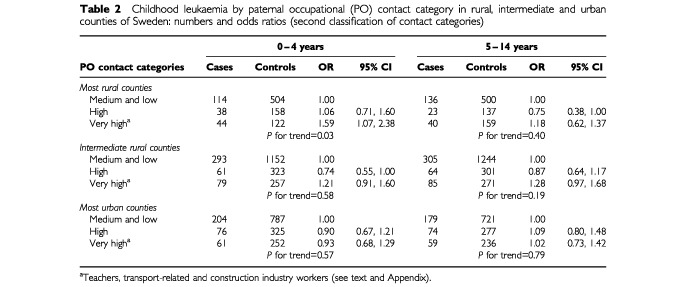
Childhood leukaemia by paternal occupational (PO) contact category in rural, intermediate and urban counties of Sweden: numbers and odds ratios (second classification of contact categories)

. No such excess or trend was observed in the intermediate or urban counties at these ages, or at ages 5–14 years in any of the three groups of counties (Table 2).

An examination of risk by socio-economic status in each of the three county groups showed no significant trend in any age group.

## DISCUSSION

Occupational risks of infection are well known, but less familiar is the possibility of increased risk among children of people in certain occupations. However, an increased risk of cytomegalovirus infection is well established among the children of nursery workers and of certain other child-linked groups (Stagno *et al*, 1982, 1984; Adler, 1988; Yow *et al*, 1988; Murph *et al*, 1991). Epidemics of paralytic poliomyelitis have occasioned similar observations about the children of teachers and of men in other high contact occupations (McFarlan *et al*, 1946; Cowan, 1950; Logan, 1953). Also, a study of poliovirus antibody levels in relation to social factors found that a higher than average level for an individual's social class was often associated with a high-contact occupation (Backett, 1957). The hypothesis that paternal occupational contact level might, in certain circumstances, influence the risk of childhood leukaemia is therefore plausible. This is particularly so in relation to pre-school children who, compared to older school-attending children, have a more restricted level of contacts, to which fathers must make an important contribution. In Sweden, children start primary school later than in many countries, at age 7 years, but earlier attendance in the smaller mixing units of kindergartens (from age 3 or 4 years) is common, though probably less so in rural areas. Against this background and the positive findings of two previous studies of rural Britain (Kinlen, 1997; Kinlen and Bramald, 2001), the results of the present study in Sweden are noteworthy: specifically, the significant relative excess of leukaemia in rural areas among the children aged 0–4 years with fathers in high contact occupations, as well as the significant positive trend in these areas across categories of increasing contact level.

In the recent Scottish case–control study of childhood leukaemia and paternal contact level in rural areas (Kinlen and Bramald, 2001), a question left unresolved was whether the effect was typical of very rural areas (as much of Scotland is) or whether it was influenced by population mixing associated with the North Sea oil industry. The finding of a similar effect in the most rural group of Swedish counties without such population mixing suggests that it is not a prerequisite for observing these paternal occupational effects. However, the lack of an inverse relationship at ages 5–14 years, which was noted in the Scottish study (and attributed to immunising effects) may indicate that in Sweden the effect was lower in intensity because appreciable population mixing was absent. That the risk of childhood leukaemia should vary in subgroups within situations falling well short of intense population mixing is not remarkable; it accords with the conventional view that open populations are made up of innumerable, but often interlocking, subgroups differing in proportions of immune and susceptible individuals, as well as in the intimacy of their contact, and that these differences are important in the spread of infection (Fox *et al*, 1971).

The absence of paternal occupational effects in urban areas of Sweden accords with the findings in urban areas of Scotland (Kinlen and Bramald, 2001) and of England and Wales (Kinlen, 1995) and also in (the largely urban) England and Wales as a whole (Fear *et al*, 1999). This is also consistent with the findings of the many studies of population mixing situations. Urban areas with their higher population density and consequent higher prevalence of immune individuals are relatively resistant to epidemics. In such areas, cases would tend to be sporadic in nature. In contrast, rural areas with their higher prevalence of susceptible individuals tend to be more vulnerable to infective outbreaks when subgroups within their populations experience higher than usual levels of contact with infected people.

The detection of significant occupational effects in rural areas of Sweden using each of two different contact classifications, as in the study of rural Scotland, makes chance or bias unlikely explanations. Nor can confounding by socio-economic factors explain the findings, as no socio-economic effect on incidence was found in the present study. Such confounding is also made unlikely by the persistence of the significant effects when the second classification of contact categories was applied in which the very high contact category included not only teachers but also construction and transport workers, groups differing appreciably from teachers in socio-economic status.

By choosing paternal occupation from the census closest to the year of birth, we aimed to ensure as far as possible that the paternal occupation recorded for this study preceded the diagnosis of the disease, and by at most only a few years in the case of children aged 0–4 years, the group of primary interest.

For the investigation of these rural effects, it would seem important that a very high contact level be included in the classification. The studies by Roman *et al* (1994) and Fear *et al* (1999) lacked this and also had a large low contact category, whereas the only occupational group of any size so allocated by our advisors was agricultural occupations. Applying their classification to our data would have largely missed the effects reported here.

In general, acute lymphoblastic leukaemia predominates in the 0–4 years age group, but an appreciable proportion of the rural cases in the highest contact categories in the present study were diagnosed before 1975 and had been coded simply to leukaemia of unspecified type.

The findings are the more noteworthy in view of certain limitations of the study that were conservative to the hypothesis, namely the relatively crude categorisation both of rural–urban status (whole counties) and of contact levels (based on fewer job titles) as compared with our previous studies. It is also relevant that occupation represents only one of the opportunities for fathers (and therefore indirectly for their children) to have contacts with a wider infective pool. However, no account could be taken in this study of the social and recreational contacts of either parent. To a lesser extent, this also applied to the contact level of maternal occupation which until recently was poorly recorded in the Swedish census (Mutanen and Hemminki, 2001).

These findings at ages 0–4 years over a wide (40 year) period in rural Sweden represent further support for an infective basis in childhood leukaemia. However, the negative findings in the so-called intermediate counties indicate that for the examination of risk in relation to occupational contact level, the most rural, even isolated, areas should be made the primary focus of interest. Similar studies, as well as studies of situations of marked urban–rural population mixing, may be useful in the investigation of a possible infective basis among the many cancers, and other diseases, that are of uncertain aetiology.

## Nordic occupation codes categorised by contact level

### First classification

*Low and medium contact levels* (the reference category)–All occupations not mentioned below, except for ‘economically inactive persons’ (code 54) who were excluded from the study
*High contact level*–Service and professional workers: sales workers (14,15), doctors (3), dentists (4), nurses (5, 6), other health and medical workers (7), religious and social workers (9), waiters (47), hairdressers (50), other service workers (51), military personnel (52). Transport and communication-related occupations: seamen (21), drivers (23), transport workers (22), communication workers (24). Construction industry: plumbers (29), painters (33), bricklayers (35), other construction workers (34).
*Very high contact level*–Teachers (8).

### Second classification

*Low and medium contact levels* (the reference category)–As in the first classification (see above).
*High contact level*–Service and professional workers: sales workers (14, 15), doctors (3), dentists (4), nurses (5, 6), other health and medical workers (7), religious and social workers (9), waiters (47), hairdressers (50), other service workers (51), military personnel (52).
*Very high contact level*–Teachers (8). Transport- and communication-related occupations: seamen (21), transport workers (22), drivers (23), communication workers (24). Construction industry: plumbers (29), painters (33), bricklayers (35), other construction workers (34).
